# Context and generalizability in health policy and systems research: a plea for an integrative praxis of theorizing

**DOI:** 10.1093/heapol/czaf048

**Published:** 2025-07-30

**Authors:** Sara Van Belle, Bruno Marchal

**Affiliations:** Department of Public Health, Institute of Tropical Medicine, Nationalestraat 155, Antwerp B-2000, Belgium; Complexity and Health Unit, Department of Public Health, Institute of Tropical Medicine, Nationalestraat 155, Antwerp B-2000, Belgium

**Keywords:** context, theorizing, theory-building, realist evaluation, realist research, implementation science

## Abstract

In this article, we address the conundrum of context in health policy and systems research, zooming in on research on implementation of programmes, policies, and interventions. We review how the field draws on non-linear paradigms to better take into account ‘context’ in causal explanation, and we compare paradigms and the way in which they can inform more context-sensitive research, policies, and programmes. We propose a theorizing praxis that is based on the principles of realist inquiry and that allows researchers to draw lessons applicable to other settings by integrating a comprehensive analysis of context in their research.

Key messages

- In health policy and systems research, the importance of context as a causal factor is widely acknowledged, yet the causal power of context remains a slippery methodological issue. Producing generalizable results while situating health policies, programmes, and interventions in their specific context represents a conundrum.
- Realist inquiry provides a sound frame for a theorizing praxis, as it is centred on theory development and testing. The realist approach to developing hypotheses that explicitly include ‘context’ and testing them empirically in different settings is useful, particularly when theories and frames from non-linear paradigms are used to inform the hypotheses.
- A theorizing praxis based on realist inquiry principles would enable researchers not only to assess the effectiveness of policies, programmes, and interventions, but also to explore how and under which conditions they work. Drawing upon such studies, policymakers and practitioners may be able to better assess whether the intervention would work in their context.

## Introduction

For a long time, health policy and systems researchers have been facing the challenge of doing justice to ‘context’ in research and evaluation of health policies, programmes, and services. On the one hand, the way ‘context’ shapes the observed outcomes and the implementation of an intervention needs to be understood. On the other hand, researchers need to indicate the transferability of their findings to other settings. Context specificity and generalizability are, in effect, two sides of the same coin.

Since Gilson *et al.* called attention to the issue (Gilson *et al.* 2011), the field of health policy and systems research (HPSR) has shifted slowly but steadily towards a more dynamic view on context. This shift was amplified by the decolonization of global health movement, which tasked the global health community, and in particular staff of global health research institutions that have historically been associated with colonialism, to critically reflect on the position of their institutions in global health research (Renmans *et al.* 2022, Affun-Adegbulu *et al.* 2023). Part of the critique focused on the fit of theories and concepts developed in high-income countries (‘WEIRD’ theories) to settings in low- and middle-income countries (LMICs) and on epistemic injustice (Gilmore 2019, Bhakuni and Abimbola 2021).

Yet, the analysis of context remains a slippery thing. Perhaps this should not surprise us. How ‘context’ exerts its influence on implementation of interventions and on their outcomes remains elusive (Pollitt 2013). Even the essence of ‘context’ is not clear. What is ‘context’? Is it a static background to an intervention, or should we think of it as a theatre stage where things happen and actors use stage props in a scene? Does ‘context’ interact with the ‘actors’ as much as ‘actors’ interact with ‘context’ to produce outcomes (Virtanen 2013)? Is context best considered as composed of levels or layers at different scales? If so, how are these layers conceptualized and defined, and how do they causally relate to each other (Daivadanam *et al.* 2019)? A whole set of methodological issues remain, despite its generally acknowledged importance. ‘If (context) is everything “out there”, then surely it is also nothing? We cannot get far with a concept that is so omnibus-like.’ (Virtanen 2013).

A way out of the ‘context trap’ is to pay more attention to developing and testing theories that explicitly position ‘context’ in the causal explanation (Chambers and Emmons 2024). Swedberg pointedly made the case for theory-building as a praxis in social science, an activity and craft that HPSR researchers are well-placed to adopt (Swedberg 2014a, 2014b). This would allow the field to move away from pragmatic ‘bricolage’ of theories and frameworks in the field of HPSR (Jones *et al.* 2021). We argue that researchers who work within non-linear paradigms are well placed to contribute to an integrative praxis of theorizing in the field of HPSR.

## What have non-linear paradigms to offer?

Non-linear paradigms have been slowly percolating into the field of HPSR even if epistemological diversity is still lacking (Ramani *et al.* 2023). Below, we focus on the commonalities and differences in how ‘context’ is considered in interpretivism, critical theory (CT), critical realism, realist evaluation, complexity theory, and pragmatism. Our aim is not to provide an in-depth overview but to show how researchers adopting these paradigms could contribute to developing a theorizing praxis to enhance causal explanations that meaningfully include ‘context’ on the basis of their paradigm-specific positions.

Interpretivists locate their studies in the specific context of the subjects and focus on the discrepancies between discourse and actual practice. They adopt the ‘emic’ perspective, aiming at developing an understanding of how people experience their realities, and develop identities and social practices situated within their context. The emphasis is on how context-specific interpretations, shared meanings, and norms shape the practices of actors. Immersion of the researcher in the study’s setting and observation are preferred methods. In HPSR, interpretivist studies examined, for example, the influence of global actors on national-level health policy processes in LMICs. MacDonald (2022), Shiffman (2007), and Shiffman and Okonofua (2007) focused on agenda setting and framing in maternal health policy development. Shiffman and colleagues situated Shiffman and Okonofua (2007) national-level policymakers in their political context and explored how the latter shaped choices and practices of the policymakers. More recently, Godziewski and Rushton (2024) analysed the global and regional policy discourse related to pandemic preparedness, and Wernli *et al.* (2017) zoomed in on the global discourse on antimicrobial resistance.

Generalizability has never been a primary concern of interpretivists, in whose view there is no unmediated access to a reality independent of the researcher (Schwartz-Shea and Yanow 2012). From their point of view, findings of small studies (i.e. where *n* is a small number) cannot be generalized (Timmermans and Tavory 2012). More recently, however, interpretivism is experiencing a ‘causal turn’ (Abell and Engel 2023). It is argued that findings from interpretivist studies can be generalized when the same shared meaning or interpretation is found in a group (e.g. faith-based motivation of medical professionals) or in different settings (e.g. competitive research cultures in American and European universities). In other words, researchers can identify context conditions under which the results from interpretivist studies hold: ‘*When context is taken into account in research, the study findings are more likely to indicate the conditions under which evidence does or does not generalize to different populations, settings, and time periods*.’ (Chambers and Emmons 2024). In the field of International Relations, Norman (2021) proposed that interpretivists can develop stronger causal explanations by not just describing the social systems in which actors are situated, but by developing an understanding of how these social systems contribute to the observed changes. ‘*The causal explanations of social action require identification of the causal capacities of the social system in which agents act. Social wholes, such as organizations, systems of rules, norms, and taken-for-granted social practices, can be thought of as assemblages of causal capacities that will shape the effects that particular events will produce*.’

CT comprises a very wide range of approaches, from the Marxist Frankfurter Schule and poststructuralism to feminist theory, postcolonial studies, and intersectional theory. Since we cannot cover their differences meaningfully in this paper, we will just indicate how principles of CT can inform context-sensitive explanations. Critical theorists share a common purpose of exposing the influence of hidden power dynamics and internalized macro-structures, such as race, religion, ethnicity, poverty, and class (Romero 2018). Their intention is to unveil these structures and to attribute causal powers to such social structures, which are seen as systems of oppression. Critical theorists call, for instance, for assessing how historically rooted context factors, such as colonialism, women’s oppression, racism, or religious discrimination, shape not only knowledge production but also policies, programmes and interventions, and their outcomes. CT has only gradually been taken up in the field of HPSR, e.g. by way of Foucauldian approaches to discourse and power.

While critical realism, scientific realism, and realist evaluation are different in some epistemological and methodological aspects, they share the view that outcomes of programmes are caused by generative mechanisms that underlie implementation processes (van Belle and Contractor 2017, Sarkies *et al.* 2022). In other words, causal explanations of outcomes need to be grounded in the interaction between agency and context. It should be noted that it is not only realists who use mechanism-based explanations: a mechanism-based approach has long been a staple in political science (e.g. Tilly 2001) and sociology (e.g. Elster 1989 ). All three realist ‘schools’ also hold that through comparative research, theories of the middle range—that encompass different contexts and settings—can be developed. Archer’s structure–agency–culture framework is a useful complement to the analysis of context (Pawson 2013) as it allows the identifying of causal powers in contexts beyond the time and space of the intervention (Van Belle *et al.* 2023).

Complexity theory entered into health care research around 2000 (Plsek and Greenhalgh 2001). Its adherents consider processes and outcomes in health policy and systems to be dynamic and emerging from the interaction between intervention, actors, and context (de Savigny and Adam 2009). These interactions are essential components of the complex adaptive system in which actors and interventions are embedded. Collective action and individual agency interact with context to actively shape emergent outcomes. Outcomes are considered to be multi-determined, and success is evaluated in terms of contribution of the intervention to the outcome and not attribution of outcomes to the intervention. The concept of implementation fidelity is replaced by the need for context adaptation and ‘elasticity’ (May *et al.* 2016). Complexity theory is also putting the role of time and space dynamics back on the research agenda, as it considers that the emergence of actions and outcomes in time and space is at the core of causal processes in complex systems (Byrne and Callaghan 2014).

A last paradigm we would like to introduce is pragmatism, which focuses on producing action-driven, practical knowledge and co-created inquiry (Barthe *et al.* 2013, Brown and Tavory 2024). Together with interpretivism, pragmatism emphasizes the construction of knowledge in action. Meanings are actively negotiated by actors during the time of the action. Pragmatism has a long history. It shaped, for instance, medical sociological studies of the negotiated order between the medical professions, such as Becker’s seminal 1961 study Boys in White (Lacqueur 2002, Nugus 2019). Pragmatism underlies the current emphasis on co-creation and public engagement in policies and programmes (Pérez Jolles *et al.* 2022, Blomgren *et al.* 2023, Longworth *et al.* 2024), as well as the use of lay or patient knowledge and expertise as a legitimate and even required source of knowledge. However, this paradigm may have the least to offer in terms of how to deal with context in causal explanations, other than its legitimization of situated or negotiated knowledge.

## Bringing the strengths of non-linear paradigms under one big ‘theorizing’ tent

As we mentioned in the introduction, strong arguments have been presented in favour of better accounting for context in HPSR, but also of developing and testing theories and theoretical frameworks (Gilson *et al.* 2011, Van Belle *et al.* 2017a). We argue that a theorizing praxis would address both concerns, but what would that look like?

In the field of implementation science, Kislov *et al.* (2019) propose that a theorizing practice would need to cover three dimensions: (i) empirical data should be approached in a theoretically informative way; (ii) the dynamic relationships between interventions, implementers, and contexts should be theorized (or causally explained) through mechanism-based explanations; and (iii) a broad range of theories from relevant disciplines should be used to inform mid-range theorizing.

In other words, researchers should develop hypotheses about how the intervention is supposedly working on the basis of theories and test these in empirical studies to assess how and in which context they work. Gradually, after repeated studies, the necessary context conditions for the intervention to be effective would be confirmed and the initial theory refined until it reaches the level of a theory of the middle range (Wang *et al.* 2023).

We propose that such an approach to theory-building can be based on the principles of realist inquiry. In his latest book, Ray Pawson, who developed realist evaluation with Nick Tilley, presents realism as a theory-building meta-frame (Pawson 2024). As we will illustrate below, the steps taken by realist researchers are well aligned with the above-described approach to theorizing. Our point is that realist research can gain a lot at each step of the realist research cycle if carried out by multi-disciplinary teams. We illustrate this with a hypothetical study on how an urban health system can be made more resilient to heat waves.

### An illustration

Let us imagine a study that aims at understanding how the resilience of an urban health system to heat waves can be improved. Central to realist evaluation is theory development and testing: all realist studies start with an initial programme theory and end with a refined theory. After repeated cycles of theory testing through empirical research, the level of a middle-range theory would be attained. This corresponds to the third point raised by Kislov *et al*. (2019) about the need for developing middle-range theories on the basis of knowledge and theories from a wide range of disciplines. In practice, realists would elicit the initial programme theory of resilience of urban health systems to heat waves on the basis of a literature review, interviews with key actors, and a (policy) document review. The latter could include municipal policies and strategies focusing on resilience, heat wave plans, and more generally emergency preparedness strategies and crisis management procedures. The analysis would focus on how the policies and interventions are assumed to contribute to resilience and under which conditions. Interviews with key respondents would help to elicit what Pawson and Tilly (1997) call the ‘folk theories’ or the assumptions held in this case by municipal health authorities, health providers, communities, elected city officials, etc. Here, an interpretivist focus on situated meaning and a pragmatist view on lay and co-created knowledge would be useful, as this would facilitate understanding of how these actors look at heatwaves, resilience, and the role of health services from their position within their context. Critical theorists would include respondents from specific groups in society that are typically excluded from decision-making processes or from interventions. As a result, a wide range of actors would be interviewed, representing a wide range of social, cultural, economic, political, and geographical backgrounds. Finally, the literature review could adopt the realist synthesis method to identify the mechanisms and context conditions required to increase resilience to heatwaves. Alternatively, a meta-narrative review could be carried out to identify the contested meanings of the concept of ‘resilience’ by examining how the concept evolved in the fields of social-ecological systems theory, psychology, critical geography, engineering, etc.

Findings of all three sub-studies would inform the formulation of the initial programme theory (IPT) of how urban health services could be made more resilient to heatwaves. One could imagine an IPT that focuses on adaptation of the infrastructure of health facilities to make them heat resistant, adaptation of the current offer of health services to better deal with surges in hospitalization of people suffering from heat exhaustion and heat stroke, and adaptation of the public infrastructure to provide shelter for people without adequate cooling at home. Alternatively, it could focus on adaptive governance arrangements, and how governance arrangements shape the absorption, adaptation, and transformation capacity of the urban health system.

Once the initial programme theory is developed, its empirical testing can start. Kislov *et al*. (2019) called for the collection of data to be steered by the initial theory and for the data that are collected to be relevant for theory development. This is fully in accordance with realist principles. Realist Evaluation is method-neutral in the sense that any data collection method can be used as long as the resulting data can be used to test the initial programme theory. If we would test the IPT of adaptive governance, for instance, the empirical data collection could include: a mapping of the current governance arrangements; an assessment of the current levels of resilience; in-depth interviews with key respondents from the health service, municipal, and national level; and an assessment of the adequacy of the governance arrangements in terms of facilitating absorption, adaptation, and transformation in relation to heatwaves.

The other point made by Kislov and colleagues was about the need to develop mechanism-based explanations that connect interventions, implementers, and context. In realist research, this configurational approach to causation is expressed by the context–mechanism–outcome configuration or its variants (De Weger *et al.* 2020). Realists aim at explaining observed outcomes in terms of mechanisms that are triggered in actors by interventions in specific contexts. However, not only ‘context’ but also the concept of ‘mechanism’ has led to much debate in realist circles. Some realist researchers find inspiration in the work of pragmatist thinkers like Hedström and Swedberg (1996), who situated mechanisms primarily at the cognitive level. In contrast, complex system adherents would consider context as the embeddedness of any actor or organization in a larger multi-level system and mechanisms as emerging from the interaction between actors and between actors and their context. Critical theorists would possibly emphasise the features of social structures that shape individual agency and collective action.

The final stage of a realist research study consists of comparing the causal explanations with the initial programme theory and refining it. Repeating studies in different contexts allows for a gradual accumulation of findings and an ever more refined programme theory that would eventually reach the level of a middle-range theory (Pawson and Tilley 1997).

More details on these principles can be found in work by Pawson and Tilley (Pawson and Tilley 1997, Pawson 2008, 2013, 2024, Pawson and Manzano-Santaella 2012). Over the last 20 years, the body of research and methodological guidance for realist evaluation and research has grown considerably. Examples of realist studies in the field of HPSR are discussed by Van Belle *et al.* (2017b) and Marchal *et al.* (2018).

## Conclusion

**Figure 1.**
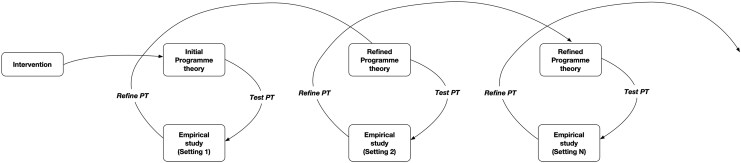
Meta-framework for theory building inspired by the realist cycle. PT, programme theory.

We acknowledge that a realist meta-frame approach to theorizing (see Fig. 1 above) may not speak to all health policy and system researchers. However, we believe that its central element of developing and testing causal explanations and theories that include ‘context’ may not only inspire researchers working in the paradigms we discussed above to develop better ways to address the causal role of context, but also stimulate them to work with realist researchers in order to develop better realist research. Whatever the resulting theorizing praxis, we argue it would enable health policy and practice researchers to better address the conundrum of context.

## Data Availability

All sources used for this study are available in public repositories with DOI identifiers.

## Reflexivity statement

This work is based on years of collaboration on methodological development in HPSR in the Department of Public Health at the Institute of Tropical Medicine, Antwerp, Belgium. S.V.B. is a female senior researcher. B.M. is a male professor. The gender balance reflects a gender imbalance in academic leadership in Belgium, where women with children have a more difficult time reaching senior academic positions. Both authors come from and now live in a high-income country, although they have both lived and worked in LMICs for extended periods. Both authors are engaged in post-Master education programmes, including master in public health and doctoral training, in which mainly students from LMICs participate. As a social scientist (S.V.B.) and public health specialist (B.M.), the authors see it as an important task to be boundary spanners, building bridges between innovative social science methodologies and methodologists and translating these to an interdisciplinary LMIC audience through publications and training.
